# Endovascular repair using a covered stent for a ruptured infected aneurysm of the superior mesenteric artery after pancreaticoduodenectomy: a case report

**DOI:** 10.1186/s40792-020-01047-w

**Published:** 2020-10-19

**Authors:** Tokuyuki Yamashita, Kenya Yamanaka, Ai Izumi, Jun Matsui, Makoto Kurimoto, Hikaru Aoki, Jun Tamura

**Affiliations:** Department of Surgery, Hyogo Prefectural Amagasaki General Medical Center, 2-17-77, Higashinaniwa, Amagasaki, Hyogo Japan

**Keywords:** Superior mesenteric artery, Ruptured infected aneurysm, Covered stent, Pancreaticoduodenectomy, Pylephlebitis, Pancreatic ductal adenocarcinoma

## Abstract

**Background:**

Delayed arterial hemorrhage after pancreaticoduodenectomy is a life-threatening complication. There are no reports about infected aneurysms of the superior mesenteric artery after pancreaticoduodenectomy without clinically relevant pancreatic fistula.

**Case presentation:**

A 78-year-old woman with borderline resectable pancreatic ductal adenocarcinoma involving the superior mesenteric arterial nerve plexus underwent pancreaticoduodenectomy with en bloc resection of the superior mesenteric vein and the superior mesenteric arterial nerve plexus after neoadjuvant chemotherapy. On postoperative day 14, she had bacteremia and sudden fever with chills. During the postoperative course, macroscopic abscesses or distinct infectious signs, including pancreatic fistula or bile fistula, were not present, but pylephlebitis was observed. After the antimicrobial treatment course, the patient was discharged. After 17 days, she was hospitalized for melena. Contrast-enhanced computed tomography showed a ruptured aneurysm of the superior mesenteric artery into the small intestine without a major intraabdominal abscess. *E. coli* was isolated from blood cultures. The patient was diagnosed with a ruptured infected aneurysm of the superior mesenteric artery. She was treated successfully with a covered stent by the cardiology team. There was no recurrence of bleeding at the 4-month follow-up, and the stent was patent in all subsequent computed tomography scans.

**Conclusions:**

Endovascular repair using a covered stent was effective in palliating acute bleeding from an infected aneurysm of the superior mesenteric artery.

## Background

Delayed arterial hemorrhage after pancreaticoduodenectomy (PD) is a life-threatening complication. Postoperative bleeding occurs in 7.6–8.5% of patients and is associated with mortality rates as high as 30% [1–3]. Delayed postoperative hemorrhage (DPH) is uncommon compared to early postoperative hemorrhage. However, it is a serious complication during the postoperative period and carries a high mortality [4]. DPH often results from inflammatory vascular erosion related to pancreatic juice or bile leakage. Although there are several reports of postoperative pancreatic fistula (POPF)-related superior mesenteric artery (SMA) aneurysms, there are no reports of infected aneurysm rupture of the SMA after PD without clinically relevant POPF or biliary leakage [5, 6]. We describe a case of successful endovascular repair using a covered stent for a ruptured infected aneurysm of the SMA after PD.

## Case presentation

**Fig. 1:**
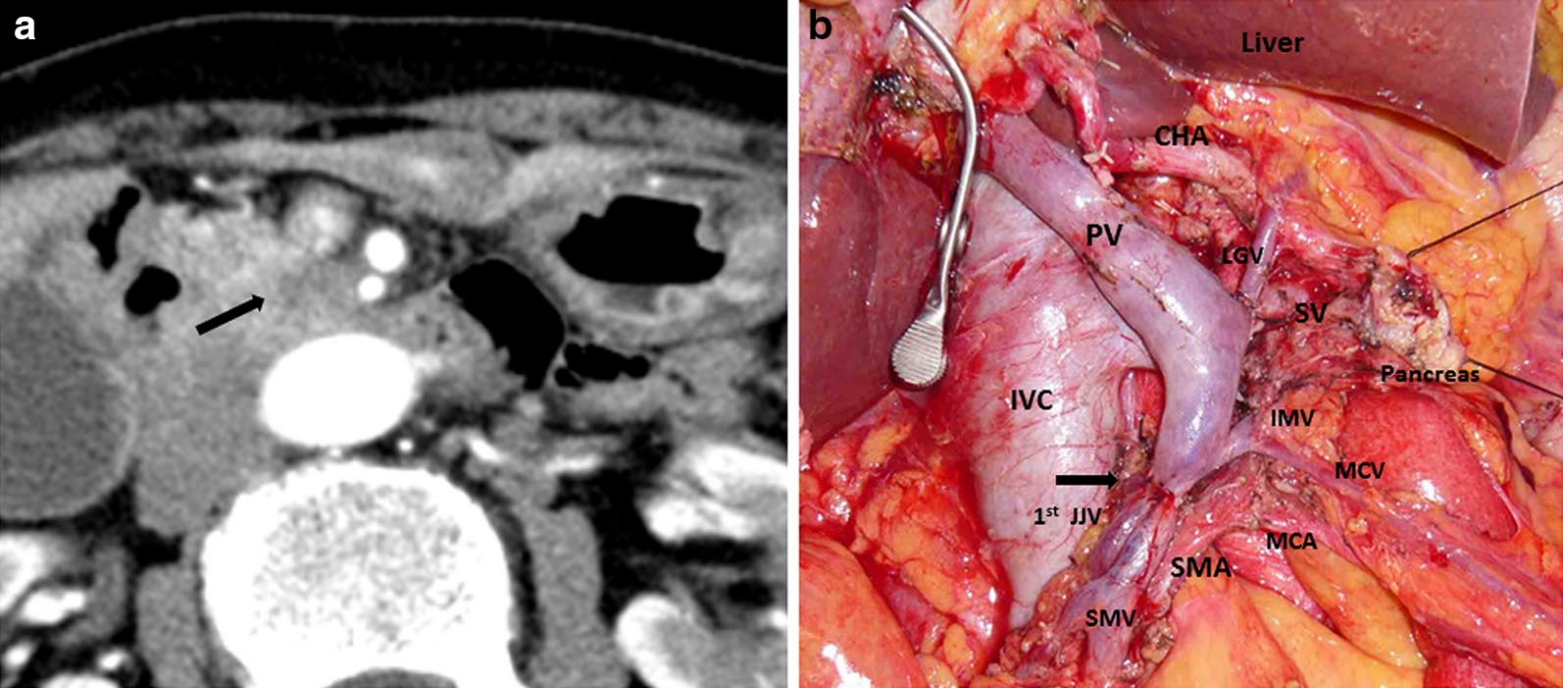
**a** Contrast-enhanced computed tomography (CECT) scan showing a hypovascular tumor abutting the superior mesenteric artery (SMA) and involving < 180° of the circumference of the artery without encasement. **b** Intraoperative photographs. The black arrowhead shows the anastomosis of the superior mesenteric vein with the 1^st^ jejunal vein. SMA: superior mesenteric artery. SMV: superior mesenteric vein. 1st JJV: 1st jejunal vein. PV: portal vein. MCA: middle colic artery. MCV: middle colic vein. IMV: inferior mesenteric vein. SV: splenic vein. LGV: left gastric vein. IVC: inferior vena cava

**Fig. 2:**
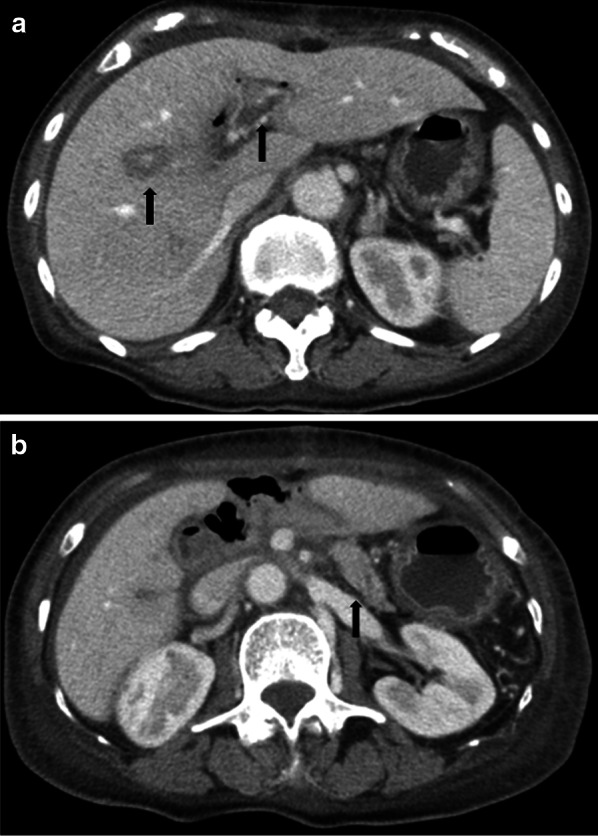
Postoperative contrast-enhanced computed tomography (CECT) evaluation. **a** CECT on postoperative day 14 shows intrahepatic portal thrombosis (black arrow) and edema of the Glissonian pedicle, while the superior mesenteric vein anastomosis was patent without thrombosis. **b** No evidence of intraperitoneal infection, such as pancreatic fistula or bile fistula, was observed. The black arrow indicates the remnant pancreas

**Fig. 3:**
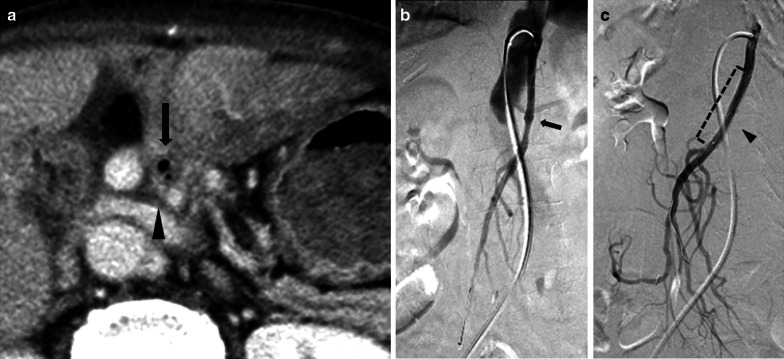
Evaluation using axial equilibrium phase contrast-enhanced computed tomography (CECT). **a** Axial equilibrium phase CECT shows the collection of gas (black arrow), an aneurysm of the superior mesenteric artery (black arrowhead) and the stomach filling with contrast fluid. **b** Superior mesenteric arteriography shows an aneurysm (black arrow), but extravasation was uncertain. **c** Arteriography after endovascular repair shows resolution of the aneurysm and preserved SMA blood flow through the covered stent (black arrowhead and dotted line)

A 78-year-old woman presented to our department with borderline resectable pancreatic ductal adenocarcinoma involving the SMA nerve plexus (Fig. 1a). She underwent pancreaticoduodenectomy with en bloc resection of the superior mesenteric vein and the SMA nerve plexus after neoadjuvant chemotherapy (Fig. 1b). On postoperative day (POD) 3, the amylase content was less than three times the upper limit of the normal serum value. Therefore, the intraperitoneal drainage tube was removed. On POD 14, she developed sudden fever with chills. The white blood cell count was 3,100 μ/L with 89% neutrophils, and the serum C-reactive protein level was 3.43 mg/dL. Contrast-enhanced computed tomography (CECT) showed intrahepatic segmental portal vein thrombosis without macroscopic abscesses or distinct infectious signs, including pancreatic fistula or bile fistula (Fig. 2a, b). After blood specimens were obtained for culture, tazobactam/piperacillin was administered. Since the blood cultures were positive for *Enterococcus faecium*, tazobactam/piperacillin was switched to vancomycin. We started novel oral anticoagulants for portal vein thrombosis on the day of diagnosis. When antithrombin III activity decreased, we added it to the drug regimen. On POD 19, *Enterobacter cloacae* and *Klebsiella pneumoniae* were recovered from the blood cultures, and meropenem was additionally administered. On POD 27, the antimicrobial treatment regimen was switched to levofloxacin based on the microbiological results. On POD 29, intrahepatic portal thrombosis had decreased, and the patient was discharged on POD 37. On POD 54, the patient presented with melena and was hospitalized for gastrointestinal endoscopy. On POD 55, she experienced cardiopulmonary arrest caused by hemorrhagic shock due to melena. Cardiopulmonary resuscitation was successfully performed. CECT showed a ruptured SMA aneurysm and arterio-intestinal fistula (Fig. 3a). Blood was lost into the reconstructed jejunal limb in the main SMA. No major intraabdominal abscess due to pancreatic leakage or biliary fistula was observed. The white blood cell count was 5,400 μ/L with 86.1% neutrophils, and the serum C-reactive protein level was 2.98 mg/dL. Thus, she was diagnosed with a ruptured aneurysm of the SMA. Since the laparotomic approach seemed difficult after the PD operation with resection of the SMA nerve plexus, we selected a covered stent as the preferred treatment option. Repair using the covered stent was performed by the cardiology team. This treatment successfully stopped the intestinal bleeding, and the patient recovered from hemorrhagic shock (Fig. 3b, c). *E. coli* was isolated from arterial blood cultures during angiography. Finally, she was diagnosed with a ruptured infected aneurysm of the SMA. After a 6-week course of intravenous antibiotic therapy, she was switched to long-term oral amoxicillin and clavulanic acid and was discharged on POD 100. There was no recurrence of bleeding at the 4-month follow-up, and the stent was patent in all subsequent CECT scans. Unfortunately, the patient died 7 months after the initial operation due to disease progression with multiple liver metastases.

## Discussion

DPH after PD is defined by the International Study Group of Pancreatic Surgery (ISGPS) as hemorrhage occurring > 24 h postoperatively. In two previous studies, DPH was defined as postoperative hemorrhage after 2 weeks and postoperative hemorrhage after 7 days [1, 7, 8]. Delayed bleeding is the result of multifactorial pathogenesis including an association with other pancreatectomy-specific complications, such as postoperative pancreatic fistula and infection [9]. The celiac artery is the common site of delayed bleeding, and the gastroduodenal artery stump is the most common location (29%), followed by the common hepatic artery (19%), the splenic artery (12%), and the proper hepatic artery (7%). However, bleeding from the SMA is rarely encountered [10]. POPF is considered to be associated with late hemorrhage. Leakage of enzyme-rich fluid into the abdomen might cause vessel erosion, which can result in the formation of a pseudoaneurysm. The pseudoaneurysm may rupture, resulting in late hemorrhage [1].

Since the development of the ISGPS classification, interventional drainage has become the standard care for patients with symptomatic postoperative fluid collection or undrained POPF [11]. The POPF diagnostic criteria by the ISGPS are as follows. Any measurable volume of drainage fluid on or after postoperative day 3 with an amylase level > 3 times the upper limit of normal amylase for each specific institution is the necessary threshold. To be defined strictly as POPF; however, this condition needs to be clinically relevant [12]. Regarding POPF-related SMA aneurysms, this case had no pancreatic leakage according to the ISGPS definition. On postoperative CECT, there were no distinct fluid collections that could be diagnosed as pancreatic juice leakage on the image. Thus, we determined that there was no clinical pancreatic leakage.

On the other hand, the SMA is the most frequently involved artery in cases of infected aneurysms, and the hepatic artery is the second most common site among the splanchnic arteries [13–15]. Infected aneurysms are diagnosed by a clinical diagnosis of infection, the identification of an aneurysm, and positive cultures from either blood or tissue. In addition, arterial blood culture is necessary [16]. Primary infected aneurysms arise from adjacent surrounding areas of infection or trauma, either from direct contact or via lymphatic spread. Secondary infected aneurysms arise from septic embolization, either through the vasa vasorum or intraluminally into areas of abnormal intima, such as preexisting aneurysms, atherosclerotic plaques, or trauma. Characterization of the causative bacteria is important, since gram-negative sepsis results in higher rupture rates than infection with gram-positive bacteria [17]. Although infective endocarditis (IE) is the most common cause of infected aneurysms [13], IE was not observed in the present case.

In the present case, there was no evidence of POPF or macroscopic abscess on CECT. However, evidence of fever spike, bacteremia, and intrahepatic portal vein thrombosis was compatible with cholangitis. Septic thrombophlebitis of the portal venous system is often associated with an infectious source in the gastrointestinal tract and sepsis [18]. Biliary infection or thrombophlebitis may spread via the portal venous system. Cholangitis might induce arterial flow in the Glissonian pedicle, which might relatively reduce portal flow. Adjacent biliary inflammation might influence portal stenosis or reduced flow due to the thin venous wall and low venous flow, which may result in intrahepatic portal vein thrombosis [19]. We suspect that the present patient may have suffered from intrahepatic portal vein thrombosis secondary to cholangitis after choledochojejunostomy and an infected pseudoaneurysm of the SMA caused by continuous pylephlebitis. Considering the pathogenesis of infected pseudoaneurysms of the SMA, follow-up blood cultures are necessary, and antibiotics should be administered for more than 4–6 weeks until complete resolution or cavernous transformation of the thrombus in accordance with the recommended treatment duration for pylephlebitis to prevent infected aneurysms of the SMA [20, 21].

It is possible that the SMA wall had weakened due to dissection of the SMA peripheral plexus. Since tissue from the SMA was not taken, it is not completely known whether the cause was from the adventitia side of the SMA or from the intimal side of the SMA. In addition, unruptured aneurysms were not observed in the CECT images until the ruptured aneurysm was confirmed, although slight injury of the adventitia of the SMA cannot be completely denied.

Because blood cultures were not regularly followed up after the pylephlebitis, it is impossible to completely show how detected enterobacterium in blood stream changed from enterococcus faecium to *E. coli*. We consider that it was important to perform blood culture follow-up and long-term antibiotic administration for avoiding infectious complications, and it is an important issue to clarify causative pathogenesis to overcome infectious disease after the PD operation.

Sentinel bleeding is relatively common in patients with postpancreatectomy hemorrhage, with an incidence ranging from 14.8 to 71.4%. However, a small amount of sentinel bleeding can result in disastrous arterial hemorrhages, which mainly stem from abdominal artery pseudoaneurysms or vessels [22]. Early recognition and familiarity with the management options are critical due to the high rupture rate and the high mortality rate associated with pseudoaneurysm rupture [8]. In the present case, we believe that hospitalization at the first incidence of melena proved to be lifesaving.

DPH has been preferentially treated with endovascular techniques, although some groups advocate operative intervention [23]. We thought that the surgical approach to the arterio-intestinal fistula was extremely difficult, as the operative field after PD with portal vein reconstruction is hostile. Transcatheter arterial embolization is usually performed for bleeding from pseudoaneurysms such as those at the gastroduodenal arterial stump. However, this method is not suitable for bleeding from the main SMA, as blood flow in this vessel must be preserved to prevent bowel ischemia. Therefore, endovascular repair using covered stents is a useful option, although it requires the radiologist to be knowledgeable about interventional radiology. Complications after endovascular management of postpancreatectomy hemorrhage have been described in the literature. These include rebleeding, stent stenosis or thrombosis, and infarction of end organs secondary to thrombosis and embolization [24]. Although endovascular repair using covered stents for infected pseudoaneurysms has been reported to be effective in controlling acute bleeding, there is always a risk of long-term graft infection. The indications for stent-graft deployment in infectious pseudoaneurysms remain controversial. Endovascular repair using covered stents is effective in palliating acute bleeding from the SMA. However, more data are needed to evaluate the long-term prognosis [25].

## Conclusions

Endovascular repair using a covered stent was effective in palliating acute bleeding from an infectious aneurysm of the SMA. Long-term antibiotic administration and follow-up blood cultures are necessary in cases of pylephlebitis after PD.

## Data Availability

Not applicable.

## Abbreviations

PD: Pancreaticodudenectomy
DPH: Delayed postoperative hemorrhage
POPF: Postoperative pancreatic fistula
SMA: Superior mesenteric artery
POD: Postoperative day
CECT: Contrast-enhanced computed tomography
ISGPS: The International Study Group of Pancreatic Surgery
IE: Infective endocarditis
